# The Effect of Studying a Double Degree in the Psychophysiological Stress Response in the Bachelor’s Thesis Defense

**DOI:** 10.3390/ijerph19031207

**Published:** 2022-01-21

**Authors:** Ana Ramírez-Adrados, Valentín E. Fernández-Elías, Silvia Fernández-Martínez, Beatriz Martínez-Pascual, Cristina Gonzalez-de-Ramos, Vicente Javier Clemente-Suárez

**Affiliations:** 1 Faculty of Sport Sciences, Universidad Europea de Madrid, Villaviciosa de Odón, 28670 Madrid, Spain; ana.ramirez@universidadeuropea.es (A.R.-A.); valentin.fernandez@universidadeuropea.es (V.E.F.-E.); silvia.fernandez@universidadeuropea.es (S.F.-M.); beatriz.martinez@universidadeuropea.es (B.M.-P.); cristina.gonzalez2@universidadeuropea.es (C.G.-d.-R.); 2 Grupo de Investigación en Cultura, Educación y Sociedad, Universidad de la Costa, Barranquilla 080002, Colombia

**Keywords:** double degree, autonomic modulation, heart rate variability, stress, university, physiotherapy academic achievement

## Abstract

The aim of this research was to analyze the effect of studying a single or double degree in the psychophysiological stress response and academic performance of university students in their bachelor’s thesis defense. We analyzed the autonomic stress response, cortical arousal, subjective distress perception, and the sense of objective and subjective academic fulfilment of 84 single-degree physiotherapy students and 26 double-degree sport sciences and physiotherapy students during their bachelor’s thesis defense. The results showed that the bachelor’s thesis defense was a stressful event for double-degree students, showing an activation of the sympathetic nervous system and presenting a higher autonomic habituation response for the double degree students compared to the single degree students. We found higher mean grades during the whole degree and higher grades in the written and oral bachelor’s thesis academic achievements for single-degree students compared to double-degree students. No significant differences were found between single-degree and double-degree students in subjective distress perception and cortical arousal. No correlation was found between academic performance variables and subjective distress perception, cortical arousal, and autonomic modulation variables. We conclude that the bachelor’s thesis defense produces a large anticipatory anxiety response in single-degree physiotherapy students and in double-degree sport sciences and physiotherapy students. Double-degree students showed higher levels of habituation and adaptability to the stressful event, with a better autonomic response. Academic achievements were significantly higher among single-degree students compared to the double-degree group.

## 1. Introduction

Double degrees, also known as dual, combined, or joint degrees, are undergraduate courses involving two bachelor’s degrees studied concomitantly over approximately five (for three-year degrees) or six years (for four-year degrees; e.g., law and engineering) [1]. Double degrees cover more than one disciplinary area to provide the education that graduates, employers, and society need in a complex, changing, and knowledge-intensive world. Previous research has shown how double-degree students may emerge more able to adapt their skills and knowledge to new contexts in flexible and responsive ways [2]. Many institutions are developing joint-degree programs as a response to an increasingly global job market [3].

This new education model is integrated in the new European Higher Education Area Space (HEAS), which establishes a bachelor’s thesis as a requirement for obtaining the bachelor’s degree. The bachelor’s thesis allows students to acquire basic research abilities, providing knowledge about scientific methods and their applications, before they finally defend the project in a public defense before a court of professors [4,5,6]. University students consider the bachelor’s thesis as the single-most cognitively demanding work at university, describing their feelings of anxiety and uncertainty [5,7,8]. Previous research also reported an increase in stress response during the writing of the bachelor’s thesis, as well as in other demanding academic activities, like clinical practices or an objective-structured clinical examination (OSCE) [9,10].

Previous studies in these demanding academic situations showed an increased sympathetic autonomic modulation of students, facts which are related with previous anticipatory anxiety responses to these academic events [9,10,11,12]. This stress response also produces the activation of the hypothalamic-pituitary-adrenal axis, affecting the correct neuronal functions of the prefrontal regions of the human brain that involve memory, decision making, and learning capacities [13,14]. 

The academic performance of students could be affected by this stress response [15,16,17], because this is modulated by factors related with the nutritional profile, body composition, and psychological profile of students, as well as their use of masks in the actual-pandemic situation [12,18,19,20,21,22,23,24]. Specifically, previous research found how the bachelor’s thesis produced a large anticipatory anxiety response for physiotherapy students that was not affected by factors such as nationality or the need to defend in native or non-native languages [4,5,6]. However, no previous studies have considered the effect of studying a double degree in this stress response. The longer the time spent in the university, the larger the curriculum developed for these students, which could be elements that allow these students to present a higher control perception in academic contexts, presenting a lower stress response. 

As such, we proposed the present research to analyze the effect of studying a single or double degree in the psychophysiological stress response and academic performance of university students in their bachelor’s thesis defense. The initial hypothesis was that double-degree students would present a lower psychophysiological stress response and higher academic achievements than single-degree students.

## 2. Materials and Methods

### 2.1. Participants

We analyzed 84 single-degree physiotherapy students and 26 double-degree sport sciences and physiotherapy students in the defense of their bachelor’s thesis. Participants were between 22 and 43 years old (M = 25.3; SD = 3.77), Heights were between 155 cm and 191 cm (M = 178.5; SD = 8.60), weights were between 53 kg and 83 kg (M = 72.85; SD = 9.87), and their body mass indexes were between 17.09 and 23.24 (M = 20.37; SD = 1.96). The proportions were 65.45% men and 43.63% women. Experimental procedures were explained to all the participants before the beginning of the research, specifying the right to withdraw from the study at any time, and requesting their voluntarily written informed consent in accordance with the Declaration of Helsinki (revised in Brazil, 2013) and approved by the University Ethical Committee (CIPI/18/074).

### 2.2. Design and Procedure

Both student groups defended their bachelor’s theses on the same day, in a conference room of the university, in the presence of three specialist professors in their subject area. The dissertation takes 20 minutes—10 minutes of dissertation and 10 minutes to reply to professors’ questions. The students have not received any feedback after the defense.

Distress perception and cortical arousal were measured 15 min prior to the dissertation (M1) and 15 min after the dissertation (M4). The autonomic modulation was measured in 4 moments: M1 (15 min prior to the dissertation); M2 (interval corresponding to the first fifth of the dissertation); M3 (interval corresponding to the last fifth of the dissertation); and M4 (interval corresponding to 15 min after the dissertation). The temporal duration of the interval was as follows:

M1: 0–15 min;

M2: 15–19 min;

M3: 31–35 min;

M4: 35–50 min.

The heart rate monitors were placed on the students’ chests 15 minutes before the start of the defense and removed 15 minutes after the defense ended, as previous studies reported [25].

### 2.3. Measurements and Instruments

Three professors, which were experts in the subject area, attended to the bachelor’s thesis and were responsible for evaluating the written work and oral thesis provided by the students. Academic performance was measured as the average between the written work grade and the oral defense grade. We also analyzed the mean grade as the average grade of all subjects during the total years of the degree or the double degree. Additionally, students were asked about their expected academic achievement (average between written and oral grades) before and after the dissertation.

Distress perception was evaluated by the subjective distress scale (SUDS), showing scores between 0 and 100, where zero (0) implies: “Completely indifferent and cold; does not affect me”, and one hundred (100) means: “So distressed and tense that I can’t deal with it”. It provides information based on the level of stress assessed by the individual, and which represents the cognitive relationship between the objective event and the emotional response [26]. 

The critical flicker-fusion threshold (CFFT) was analyzed to assess the cortical arousal using a Lafayette Instrument Flicker-Fusion Control Unit, model 12021 (Lafayette, IN, USA). Simultaneously presented in the viewing chamber were two light-emitting diodes (58 cd/m^2^), one for the left eye and one for the right eye. The stimuli were separated by 2.75 cm (center to center) with a stimulus to eye distance of 15 cm and a viewing angle of 1.9°. The inside of the viewing chamber was painted flat black to minimize reflection. The flicker frequency increment increased from 20 to 100 Hz until the student perceived fusion. Students had to respond by pressing a button upon identifying the fusion (ascending frequency) threshold [27]. Students were familiarized with the procedure by performing practice trials before testing. The practice was previous to the basal sample, in line with previous studies [28]. Three ascending trials were carried out. In each one, time was quantified as the amount of time that a student took for detecting the changes in the lights from the beginning of the test until the moment of pressing a button [27]. We used the critical flicker-fusion threshold (CFFT) as it has been widely used in different contexts, like sports, military, education, and pharmacy, to evaluate cortical arousal and central fatigue [18,20,27,29,30,31,32].

The autonomic modulation of the participants was analyzed by heart rate variability (HRV). A heart rate monitor attached to the chest (Polar V800, Polar, Kempele, Finland) was used to register the RR interval of the heartbeats from when it was placed, 15 min prior to the dissertation (M1), until it was removed, 15 min after the dissertation (M4). The RR series were analyzed using the Kubios HRV software (version 3.0; Biosignal Analysis and Medical Imaging Group, University of Kuopio, Kuopio, Finland). Thereafter, we assessed the subsequent HRV variables: minimum heart rate (HRmin); mean heart rate (HRmean); maximum heart rate (HRmax); the square root of the average of the sum of the differences squared between normal adjacent RR intervals (RMSSD); percentage of differences between normal adjacent RR intervals greater than 50 ms (PNN50); ratio between low- and high-frequency band (LF/HF); the low-frequency band in normalized units (LFn); the high-frequency band in normalized units (HFn); and the sensitivity of the short-term variability (SD1) and the long-term variability (SD2) of the non-linear spectrum of the HRV.

### 2.4. Statistical Analysis

Data were analyzed using the SPSS V.24.0 statistical package (IBM, Chicago, IL, USA). In the first place, Shapiro–Wilk analysis was used to test normal distribution of data. To assess data regarding HRV, SUDS, and CFFT mixed-factorial ANOVA was performed, including the between-subjects factor of studying a degree or a double degree and the within-subjects factor of time. Bonferroni post hoc analysis was performed when a significant F value (Greenhouse–Geisser adjustment for sphericity) was observed. Academic achievements variables were assessed by Student’s t-test procedure. Correlations between variables were explored through Pearson procedure. For all comparisons, a significance level of *p* ≤ 0.05 was accepted.

## 3. Results

### 3.1. Academic Achievement Results

**Figure 1:**
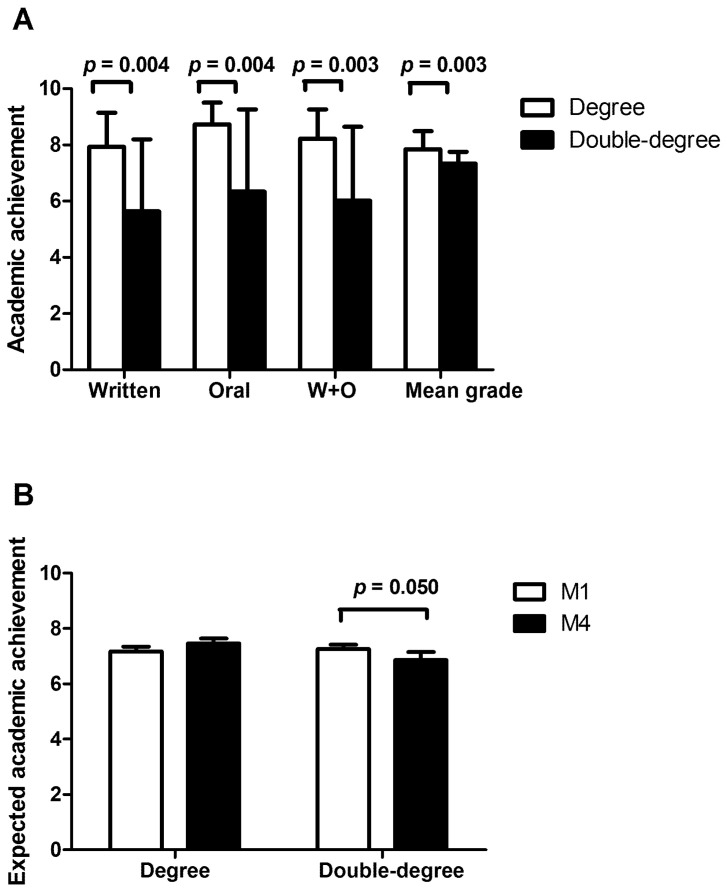
(**A**) Academic achievement (mean ± SD) of degree or double-degree students in the bachelor’s thesis written, oral, and written and oral achievement (W + O), and in the mean grade achievement; (**B**) expected academic achievement (mean ± SD) before (M1) and after (M4) in the bachelor’s thesis defense of degree or double-degree students.

We found that students enrolled in a single degree obtained significantly higher grades than those studying a double degree in the written and oral bachelor’s thesis academic achievement. Furthermore, they got significantly better mean grades during the whole degree (Figure 1A). 

### 3.2. Expected Academic Achievement Results

Regarding the expected academic achievement (Figure 1B), both single-degree and double-degree students expected similar grades before the thesis (M1). However, double-degree students expected a significantly worse grade (ANOVA F= 4.750; ANOVA *p* = 0.039; post hoc *p* = 0.050) than single-degree students after the thesis (M4).

### 3.3. HRV Results

Concerning HRV data (Table 1), a significant greater PNN50 value in double-degree students was found compared to single-degree students after the bachelor’s thesis defense. Besides this, double-degree students´ PNN50s were significantly greater in M1 and M4 compared to M2 and M3 (*p* < 0.05). No more differences were observed in the rest of HRV variables assessed. 

### 3.4. SUDS and CFFT Results

**Figure 2:**
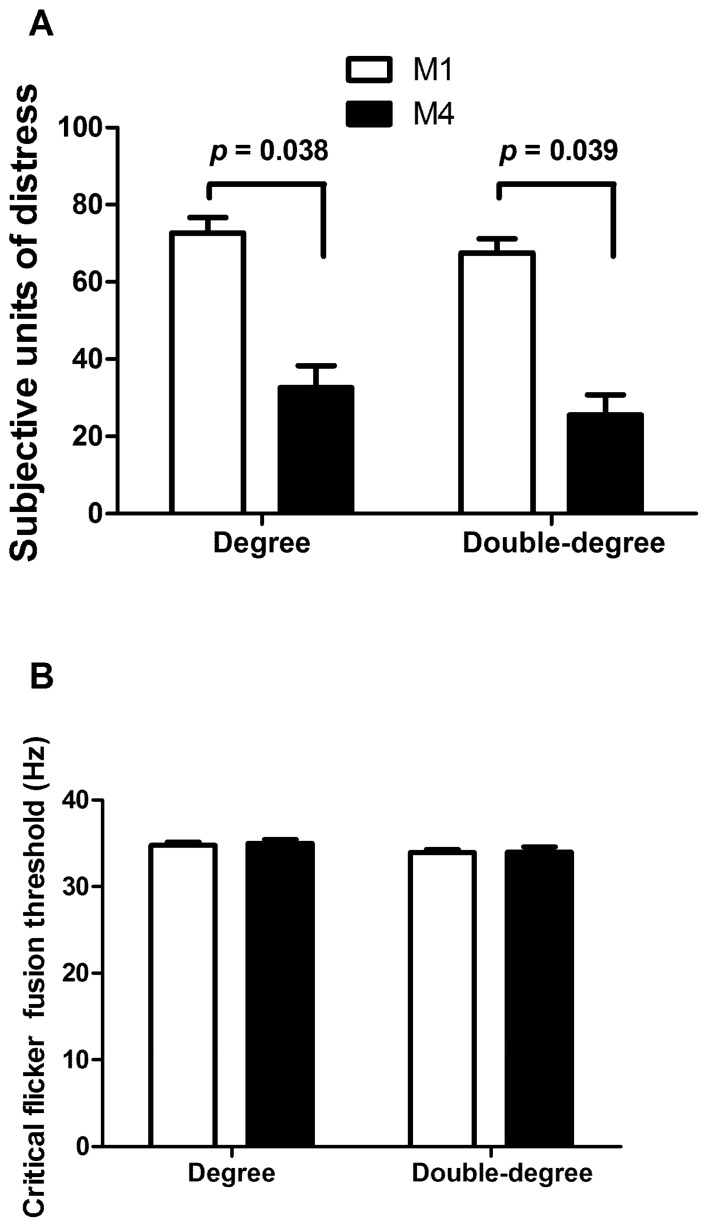
(**A**) Subjective units of distress and (**B**) critical flicker-fusion threshold (mean ± SD) before (M1) and after (M4) the bachelor’s thesis defense of degree or double-degree students.

In addition, no significant differences were found between single-degree and double-degree students in the SUDS (Figure 2A) and CFFT (Figure 2B). Nevertheless, the SUDSs were significantly lower in M4 in both groups. Finally, no correlation was found between academic performance variables, SUDS, CFFT, or HRV data. 

## 4. Discussion

The aim of this research was to analyze the effect of studying a single or double degree in the psychophysiological stress response and academic performance of university students in their bachelor’s thesis. The initial hypothesis was not confirmed since no significant differences were found in the psychophysiological stress response and academic achievements between single and double-degree students.

Results showed that the bachelor’s thesis was a stressful event for students. Regarding the autonomic stress response evaluated by the HRV results, we found how in both groups the values of PNN50, RMSSD, SD1, SD2, and HF were very low in the moment before the bachelor’s thesis defense, highlighting a decreased HRV and an increased sympathetic activity [12,33]. This anticipatory stress response has been previously studied in other academic contexts, such as the clinical stays of physiotherapy students [10] and nursing students [12], the objective-structured clinical examinations and simulation training for psychology students [9,11], the laboratory practice of pharmacy and biotechnology students [20], and non-academic contexts, such as high-level sports competition [29,34], and military combat training or parachute jumps [35,36]. All of these situations have the common factor that participants were exposed to a stressful, unknown, unpredictable, and uncontrollable situations, showing a decreased HRV, like in the present study. Exposure to uncontrolled and unknown situations causes an increased tendency in sympathetic nervous system to prepare the body to face a situation that the subject perceives as threatening [37]. Specifically, in the university context, an increased cortisol production was found in the moments preceding different types of evaluations, namely practical evaluations [38,39] affecting skills-based tasks, such as working memory, learning, or problem solving [40,41,42].

The prolonged exposure to a situation that the subject perceives as stressful can lead to an increase in HRV values and therefore a decrease in sympathetic nervous system activity. This stress habituation process has been observed in high-level sport competitions, as well as in high-intensity training, where subjects showed a habituation to these specific stressors [43]. Moreover, this habituation was also evaluated in academic events, such as in the objective-structured clinical examination for psychology students [9] showing how students´ autonomic nervous systems decreased the alert level, probably due to the decrease in the situation uncertainty and novelty, and the increase in the predictability of the situation [9].

Analyzing the habituation process of the students we found at the end of the defense event (M4) a significant increase of PNN50 in the double-degree students compared to the single-degree students. Moreover, the rest of the HRV variables showed an increased tendency, facts associated with an increase in parasympathetic modulation [44]. Double-degree students showed higher values of PNN50 at M1 and M4 compared to M2 and M3, suggesting that this group of students increased their sympathetic modulation when starting the oral dissertation, and it remained high until the stressor stimulus disappeared. We found a higher habituation in double-degree students (PNN50 in M4 12.39 ± 2.10) than single-degree students (PNN50 in M4 6.61 ± 1.51). In addition, after the defense, double-degree students recovered parasympathetic modulation faster than single-degree students, showing a more adaptative autonomic response. Furthermore, the higher stress response of double-degree students at the beginning of the dissertation could be explained by a higher self-demand from themselves, since they study a double degree, and they want to show in the defense that they are prepared [35]. Other studies regarding physiotherapy students during their bachelor’s thesis showed how sympathetic nervous system modulation remained high throughout the defense, showing low HRV values [5,6]. However, another study about the impact of nationality on the psychophysiological stress response in the bachelor’s thesis indicated that the Spanish student group showed greater parasympathetic activity from the beginning to the end of the defense, compared to the French and Italian student groups [4]. 

Previous authors showed how the HRV analysis is a useful tool to study the autonomic modulation and its effect on the learning process of students. Previous studies found high sensibility in HR, RMSSD, PNN50, LF/ HF, LF, HF, SD1, and SD2 variables to identify the autonomic modulation among physiotherapy students [5], while other studies in the objective-structured clinical examination among psychology students [9] showed sensitivity only in the frequency domain (LF and HF) and the non-linear domain (SD1 and SD2). The large individual variability in the HRV response could explain these different results. In the present research we only found significant differences of PNN50, showing for the double-degree students an increase in this value, facts which are related with an autonomic habituation to the contextual stressor. This autonomic adaption was in line with the previous research with psychology students in an objective-structured clinical examination [9] and psychology clinical stays [26], physiotherapy students in clinical practices [10], and biomedicine students in chemical laboratory practices [20]. Contrary to other academic contexts where the autonomic stress response was maintained during all the academic events, as in clinical simulation among psychology students [11], and nursing students in a clinical hospital simulation [10].

Regarding the subjective stress response, we found how both groups (single and double degree) presented large SUDS values before the beginning of the dissertation event, consistent with the increased sympathetic modulation analyzed. SUDS values decreased in both study groups after the thesis, contrarily to the maintained sympathetic arousal observed in the single-degree students’ group, but in line with the increase in the parasympathetic activity of double-degree students. These results are in line with previous studies that also observed this subjective stress-perception decrease once the stressor event disappeared, such as among psychology students performing clinical practices with simulated patients [11] or physiotherapy students from different countries in a dissertation [4].

At the end of the dissertation no significant modification in cortical arousal was found in either group. Cortical function in stress contexts has been analyzed in previous studies with stressful events showing a decrease in cortical arousal in events like mountain races, combat, ultra-endurance sport, parachuting, and high-altitude parachute jumps [13,34,45,46]. The overstimulation of cortical functions is due to the evoking environment in the case of militaries and mountain athletes, with both contexts being associated with central nervous system fatigue and subsequently presenting a large sympathetic modulation. The duration of the eliciting context in the present research, which lasted not long enough to cause this fatigue, could explain the lack of modification of the cortical arousal despite the high sympathetic modulation analyzed [35]. 

Regarding the expected academic achievements, they were similar in both groups before the beginning of the defense. However, double-degree students perceived significantly lower academic performance after the defense compared to single-degree students. These results could be explained due to the increased sympathetic modulation during M2 and M3 (stress response), and the greater self-demand from themselves.

Written and oral bachelor’s thesis achievement and mean academic fulfilment for the whole single/double degree were significantly higher among single-degree students compared to the double-degree group. It may be due to the increased student burden of having to study two degrees, which means less time to dedicate for academic credit, consequently resulting in a lower grade. Institutions developing double-degree programs should take this fact into consideration when reviewing program scheduling and student workload, so as to not be extra challenging for double-degree students.

### 4.1. Limitations of the Study and Future Research Lines

The main limitation of this study was that no measurements of hormonal stress response (cortisol, adrenaline, alpha amylase, etc.) were analyzed to gauge the adrenergic stress response of students. The number of participants in each group was not homogeneous. The single-degree physiotherapy students’ group (84 students) was larger than the double-degree sport sciences and physiotherapy students’ group (26 students). In addition, this study could be extended to other degrees, as well as to other stressful academic situations or different educational levels, such as primary and secondary schools.

Additionally, economic status was not included in questionnaires and could be considered an important issue to consider in future research, as previous authors have highlighted [47], as well as other contextual and behavioral factors, such as nutritional, odonatological, and psychological patterns, that could modulate the behavioral profile of students [48,49,50,51]. Finally, the no-analysis of anxiety response of participants could be another limitation. All of these limitations could be interesting to analyze in future studies.

### 4.2. Practical Application

The Bachelor’s thesis defense produced a large anticipatory anxiety response. The knowledge of the students´ autonomic modulations during their thesis could be useful to prepare them during the degree in order to control the psychophysiological stress response. Practical sessions preceding the bachelor’s thesis defense, would control the stress perceptions of students produced by an unknown and important academic event. Re-peated exposure to this academic act would decrease the anxiety and stress response, and increase their habituation. Other health science degrees may use this information to prepare and control the psychophysiological stress response of their students in the bachelor’s thesis defense.

## 5. Conclusions

The bachelor’s thesis defense produces a large anticipatory anxiety response in single-degree physiotherapy students and in double-degree sport sciences and physiotherapy students. Double-degree students showed higher habituation and adaptability to the stressful event with a better autonomic response. Academic achievements were significantly higher among single-degree students compared to the double-degree group.

## Figures and Tables

**Table 1:** Changes (Mean ± SD) in the autonomic stress response to the bachelor’s thesis according to whether students attended a degree or double-degree bachelor’s course.

		M1	M2	M3	M4	F-Value	*p*-Value	Post Hoc
HRmin (bpm)	Degree	78.77 ± 1.89	109.82 ± 4.05	91.46 ± 2.35	82.20 ± 2.06	1.330	0.272	
	Double degree	74.62 ± 2.56	99.04 ± 3.30	83.55 ± 2.34	72.47 ± 1.80			
HRmax (bpm)	Degree	144.53 ± 3.22	150.68 ± 6.34	127.83 ± 3.31	128.80 ± 3.66	0.764	0.475	
	Double degree	137.70 ± 3.06	137.23 ± 3.47	121.09 ± 2.54	119.19 ± 2.58			
HRmed (bpm)	Degree	110.00 ± 2.89	132.03 ± 3.56	110.50 ± 2.90	105.97 ± 2.44	0.436	0.694	
	Double degree	102.68 ± 2.66	123.68 ± 3.20	103.12 ± 2.56	95.84 ± 2.13			
RMSSD (ms)	Degree	32.13 ± 5.82	18.16 ± 5.54	19.07 ± 2.29	30.10 ± 4.34	1.115	0.347	
	Double degree	31.88 ± 3.90	18.80 ± 2.95	23.22 ± 2.60	39.86 ± 4.38			
PNN50 (%)	Degree	6.68 ± 1.80	3.36 ± 1.66	3.00 ± 0.89	6.61 ± 1.51	3.318	0.027	M4: D < DD (0.030)/DD: M1 > M2 (<0.000); M1 > M3 (0.003); M4 > M2 (<0.000); M4 > M3 (<0.000)
	Double degree	8.29 ± 1.39	3.30 ± 0.84	4.50 ± 0.87	12.39 ± 2.10			
LF/HF (n.u.)	Degree	4.27 ± 0.53	5.99 ± 0.65	6.89 ± 0.65	5.32 ± 0.62	0.810	0.473	
	Double degree	4.10 ± 0.37	4.81 ± 0.43	7.20 ± 0.76	4.34 ± 0.50			
LF (n.u.)	Degree	75.93 ± 2.36	81.02 ± 2.23	84.07 ± 1.88	79.87 ± 2.11	1.281	0.306	
	Double degree	78.28 ± 1.24	80.23 ± 1.47	86.21 ± 0.88	77.00 ± 2.03			
HF (n.u.)	Degree	24.00 ± 2.35	18.97 ± 2.20	15.89 ± 1.87	20.07 ± 2.10	1.216	0.307	
	Double degree	21.66 ± 1.23	19.73 ± 1.47	13.75 ± 0.88	22.94 ± 2.02			
SD1 (ms)	Degree	21.18 ± 4.25	10.64 ± 3.20	11.96 ± 1.28	21.21 ± 3.31	0.738	0.518	
	Double degree	23.58 ± 2.89	13.42 ± 2.22	17.36 ± 1.86	29.21 ± 3.27			
SD2 (ms)	Degree	54.73 ± 5.28	33.61 ± 5.83	45.41 ± 3.66	55.52 ± 4.74	1.309	0.280	
	Double degree	60.57 ± 4.90	41.80 ± 4.48	53.47 ± 4.44	74.05 ± 5.98			

M1: Pre-defense; M2: first 1/5 of the defense; M3: last 1/5 of the defense; M4: post-defense; HRmin: minimum heart rate; HRmax: maximum heart rate; HRmean: mean heart rate; RMSSD: square root of the average of sum of the squared differences of the RR intervals; PNN50: percentage of consecutive RR intervals that differ >50 ms; LF/HF: ratio between low- and high-frequency waves; LF; low-frequency wave; HF: high-frequency wave; SD1: variability of the short-term HRV; SD2: variability of the long-term HRV.

## Data Availability

All the data are presented in the study.
